# Exploring Why Cancer Patients Engage Into Medical Education

**DOI:** 10.7759/cureus.36358

**Published:** 2023-03-19

**Authors:** Joshua Frost, Ertan Teodorescu-Arghezi, Daniel Anderson, Lubna Bhatt

**Affiliations:** 1 Radiology and Interventional Radiology, The Christie National Health Service (NHS) Foundation Trust, Manchester, GBR; 2 Medical Education, The Christie National Health Service (NHS) Foundation Trust, Manchester, GBR; 3 Psycho-oncology, The Christie National Health Service (NHS) Foundation Trust, Manchester, GBR; 4 Oncology, The Christie National Health Service (NHS) Foundation Trust, Manchester, GBR

**Keywords:** patient reported experience, interpretative phenomenological analysis, cancer, undergraduate medical education, patient educator

## Abstract

Background and Objectives

Patients are recruited to act as educators, sharing experiences of their illness to facilitate active student learning. At our institution, cancer patient educators have been recruited to participate in a weekly teaching session for students. Our study was designed to assess the benefits that partaking in medical education confers on patients who were treated for cancer, as well as explore their motivations for becoming educators and how we can improve their experiences in the future.

Methodology

Our study used a qualitative exploratory research design, with four current patient educators being selected to participate. The interviews were conducted virtually and were designed to allow patients the opportunity to provide a rich narrative of their experiences. Their accounts were transcribed using built-in transcription software and analysed using interpretative phenomenological analysis (IPA). IPA is an in-depth analytical method used to identify common themes between patients' experiences and explore why these themes exist.

Results

Four superordinate themes, each with its subthemes were identified following analysis of patient transcripts: the perceived success of the session (relationship between patient educator and facilitator, willingness of students to participate, organisation and planning of the session), motivations for becoming a patient educator (wanting to *give something back*, personal attributes making them suitable for the role and improving experiences of future patients), perceived benefits of engaging in medical education (improvement in mental health and engaging with medical students) and suggested improvements for the session (logistics and recruitment).

Conclusions

Being a cancer patient educator offers significant benefits for patients' well-being, particularly in mental health. Cancer patient educators are motivated by the need to *give something back* to the staff and institution where they were treated. The educators also referred to improving care for future patients by educating students about negative experiences they encountered and how these could have been avoided. Finally, educators suggested improvements for future sessions by addressing the length of the sessions and having a formal recruitment process.

## Introduction

Patients have been a fundamental part of medical student education for a long time. They are recruited to act as educators, sharing experiences of their illness to facilitate active student learning. The General Medical Council (GMC) states that involving patients in medical education has a great impact on the learners as they gain communication skills and develop a professional attitude, empathy and clinical reasoning, while for the patients, it gives the satisfaction of helping, increased knowledge, confidence and self-esteem [1].

Several studies have looked into the benefits of using patients as educators and what motivates them to take this role. One study conducted semi-structured focus group discussions and individual interviews with 30 patient educators with chronic conditions who shared their experiences with students. An inductive thematic analysis of their responses revealed a majority of positive aspects, such as altruism towards the health care professionals by *giving something back*, having a sense of relief when sharing their stories and having the opportunity for self-reflection. However, certain negative aspects such as sharing too much personal information left some of the educators vulnerable, while others raised concerns regarding confidentiality [2]. The study was limited by the absence of more nuanced themes which could have emerged through more interviews and the lack of verification of the themes with the participants and the self-selection of participants who would have had a noteworthy positive or negative experience they were willing to share. Furthermore, based on the same study, the authors identified key themes regarding the messages patient educators want to instil in health care professional students. Patients want students to understand the value of inter-professional collaboration and patient-centred care and to acknowledge the long-lasting impact of chronic conditions as these will educate the students and make them better doctors in the future [3]. A descriptive study, using the structured telephone- or e-mail-based questionnaires, investigated the motivations for and experiences of 16 patient tutors living with a chronic medical condition who are taking part in a teaching workshop for medical students. The tutors were selected from a database of educators who had participated in at least one workshop. The responses from the tutors revealed an underlying altruistic motive not to *give something back* but also to teach and educate the doctors of the future by highlighting any neglected issues in their care. On the negative side, one tutor felt vulnerable discussing sensitive health topics [4]. Limitations of the study include the low number and response rate of the participants and potentially biased tutors who took part in multiple sessions for personal benefits such as a lengthier discussion with a health care professional as opposed to a standard medical consultation. A systematic review looking at the role of patient involvement in undergraduate medical education revealed patients often take the role of a teacher, sharing their health care experiences and personal aspects of their lives so that students become more confident and bring an overall improvement to the health care system [5]. Although these studies have been conducted on a small number of participants, common themes can be identified regarding why patients choose to act as educators: altruism and *wanting to give something back*, improving the experience of future patients but also making sure today’s students are better doctors in the future.

Our study involves fourth-year medical students from the University of Manchester School of Medicine who are in a one-week oncology placement at The Christie Hospital National Health Service (NHS) Foundation Trust, one of the largest cancer treatment centres in Europe. At our institution, patient educators are recruited to participate in a weekly teaching session for students. This session, known as Patient Assessment Teaching (PAT), has been run for several years. The session is a chance for fourth-year medical students rotating through their oncology blocks to listening to a patient’s journey with cancer: how they were diagnosed, what treatment did they undergo, what challenges they coped with and how they are doing in the present day. The format of the session is open, meaning students are at liberty to ask the patient any question about their experience with cancer, and it is usually facilitated by a medical professional, either a doctor or nurse specialist, who guides the students and aids them in formulating questions. Despite the session being run for several years, feedback was always aimed at students to ensure the teaching being delivered is useful and relevant to them, covering their intended learning objectives. However, we thought it would be interesting to delve into the patient’s thoughts about acting as an educator: What motivations do patients have for volunteering to be a part of medical education? What benefits do they gain from their participation? How may we improve their experience?

To assess our educators’ experiences, we have used interpretative phenomenological analysis (IPA), a methodological framework in qualitative psychology. IPA is a qualitative research process, which allows a subjective exploration of the participant’s experience from their point of view. It is a way to get insight into how an individual makes sense of a certain situation of personal significance, such as a major life event or illness, and helps researchers get access to a participant’s experience and make sense of it [6]. It was first developed and used in the field of psychology by Jonathan Smith who wanted to explore people’s perceptions of health without ignoring the individual [7]. IPA has been used in researching a variety of issues, from living with chronic back pain, and how gay men think about sex and sexuality, to how people come to terms with the death of a partner or how being HIV-positive impacts personal relationships [8-11]. There are no rules on how many participants should be included in an IPA study; however, samples are small so that each case benefits from an in-depth analysis. Data is collected is by interviewing the participants (in-depth, semi-structured or one-to-one interviews). Semi-structured interviews are preferable as there is a real-time dialogue between the participants and the researcher and can also lead to unexpected or original issues to be explored [5]. The interview questions are aimed at exploring mental phenomena such as thoughts and memories but also allowing individual interpretations and self-reflection. 

IPA has also been used in undergraduate medical education. One study looked at undergraduate medical student experience and perceptions of problem-based learning (PBL) coaching and tutoring at a Chinese medical school. Twenty students and five tutors underwent semi-structured interviews, their answers were analysed, and six main themes and sub-themes were identified regarding the students’ and tutors’ experiences in PBL coaching. The results show PBL gives students and tutors the opportunity for reflective co-learning and flexible thinking [12]. Another study aimed at exploring how peer-assisted learning (PAL) promotes learning used IPA to see how medical students from the University of Manchester experience PAL sessions. Semi-structured 30- to 60-minute interviews were conducted, and the participants were asked about their experience in PAL sessions, their reason for participation and the roles of the tutors. On further analysis of the responses obtained, it was revealed students feel PAL sessions are a safe and egalitarian environment but there is also a close student-peer tutor relationship, which promotes better learning [13]. IPA was also used in a study looking at how patients experience undergraduate medical teaching when they become engaged in it opportunistically. Ten patients, out of which nine were affected by chronic diseases such as diabetes, psoriasis or hyperthyroidism, were interviewed in a semi-structured way to find out how they felt when a student was present during their medical consultation. For all patients, the presence of students was regarded to be a normal practice as it was assumed to be beneficial for the students, thus for society overall. As professional interactions were conducted between the student and the doctor, patients felt more like outsiders and objects from which students learned rather than having an active role in students’ learning. For most patients, this was considered to be normal, but for one patient, with a background of working as a health care professional and clinical educator, this was unusual and felt alienated as she expected to actively take part in the teaching process [14].

What makes our study unique is that it focuses solely on oncological patient educators, and as far as we know, there is no other similar study documented in the literature. Our study was initially conceived as an internal service improvement project, and plans for the project were approved by our institution’s Quality Improvement Change Assessment (QICA) team.

## Materials and methods

The study used a qualitative exploratory research design. The aim was to develop a greater understanding of patient educators’ experiences volunteering within medical education, particularly regarding a teaching session delivered to fourth-year medical students rotating through their oncology block. A sample of four current patient educators was selected to participate in the study. The participants were selected from a pool of six former patients who are in remission from cancer. They have all been previously treated at The Christie Hospital NHS Foundation Trust and have taken part in at least one PAT session in the past. All patients were contacted via email and four of them responded positively. No discrimination between the patients’ gender, age, type of cancer or type of treatment was applied. Interviews were semi-structured, with questions that were agreed upon beforehand by two authors. Questions were selected that would enable perspectives on the experience of engaging with medical education during cancer to be elicited. All four participants were asked how they became educators, why they accepted this role and how it impacted them. They were also asked what benefits and challenges they encountered as patient educators and what needs to be improved for future sessions (Appendix). All patients were interviewed virtually during March 2022, using videoconferencing. Their accounts were transcribed using built-in transcription software, cross-referenced with the video interviews to ensure the accuracy of the transcription and then analysed using IPA. IPA is a qualitative methodology of psychology research and is an in-depth analytical method used to identify common themes between patients’ experiences and explore why these themes exist. Two authors independently read the transcripts to identify themes for the interviewees. The themes were then collated together to create macro themes once a consensus was reached. 

## Results

Following the interviews and data analysis, four main superordinate themes were identified, each with its sub-themes (Table 1).

**Table 1:** Superordinate themes and sub-themes.

Superordinate theme 1: Perceived success of the session	Superordinate theme 2: Motivations for becoming a patient educator	Superordinate theme 3: Perceived benefits of engaging in medical education	Superordinate theme 4: Suggested improvements for the session
Sub-theme 1.1: Relationship between patient educator and facilitator; Sub-theme 1.2: Willingness of students to participate; Sub-theme 1.3: Organisation and planning of the session	Sub-theme 2.1: Wanting to *give something back*; Sub-theme 2.2: Personal attributes making them suitable for the role (e.g., teacher, nurse and advertiser); Sub-theme 2.3: Improving experiences of future patients	Sub-theme 3.1: Improvement in mental health; Sub-theme 3.2: Engaging with medical students	Sub-theme 4.1: Logistics; Sub-theme 4.2: Recruitment

The perceived success of the session

Patient educators were all involved in delivering teaching to students as part of the PAT session. Patients’ thoughts on how *well* a session went related to several key factors, which have been split into varying sub-themes.

Sub-theme 1.1: Relationship Between Patient Educator and Facilitator

"A lot of it depends on the facilitator… so if you sort of know which are the good facilitators… although most of them were really good.” (Patient 1). 

Patient 1 felt as though the success of the teaching they were involved in was greatly influenced by the abilities of the facilitator. When asked to elaborate on why they thought the success of the session was tutor dependent, the patient answered as follows:

"[…]And some of the facilitators made me go out of the room while they're formulated questions. And I found that didn't work very well. It might work better for other people, but for me it broke up this sort of… You told your story, and then they obviously had a lot of things in their head that they wanted to ask you, but it just felt a bit fake." (Patient 1)

For students to have time to formulate questions for the patient, facilitators on occasion would send the patient out of the room. However, Patient 1 felt that this made the resulting conversations feel *a bit fake*, as there was an interruption to the flow of the discussion. Furthermore, the tutor assisting students with the content of the questions may have also contributed to the patient expressing the discussion felt fake, as it took away from the students’ freedom to ask exactly what they wanted to know, perhaps being steered towards a particular narrative by the tutor, which was picked up on by the patient.

Patient 4 also reported that the variety of facilitators impacted their experience. On one occasion, the patient described their experience as follows:

"I went and just sat there like a lemon, thinking actually there's not much point being here, they're not interested in what I've got to say." (Patient 4)

This demonstrates that there is a disparity between facilitators, which results in a mixed experience for the patient educator depending on how the session was run. It seems that facilitators that take a step back and allow the patient to lead the session are preferred by the educators, although there is a fine balance to ensure that the questions asked align with students’ intended learning outcomes.

Sub-theme 1.2: Willingness of Students to Participate

A couple of patient educators also felt as though the success of the session depended on the student's level of interaction and confidence to ask them questions. For Patient 2, they enjoyed trying to encourage participation from students:

"Whenever you've got a, uh, a crowd of people, you know it might be 20, sometimes it was 30, and you get those that are quite shy… and I think I sort of brought out their answers." (Patient 2)

Patient 2 also referred to how their previous career in advertising meant they had personal qualities, which encouraged students to "come out of their shells," perhaps also reaffirming to themselves that they were suitable for the role.

"And sometimes you got really good discussions depending on the group. Some of them are more talkative than others." (Patient 1)

"Because it takes you half an hour to break the ice with the medical students. And I think once they feel more comfortable with you, then they’ll ask those in-depth questions." (Patient 3)

The above quotes from Patient 1 and Patient 3 further exemplify the variance in student groups, with Patient 3 putting an emphasis on the time needed to talk with students to *break the ice* and allow them to become more comfortable asking personal questions to someone that they have only just met.

Sub-theme 1.3: Organisation and Planning

Most of the patients were very complimentary of the medical education staff that organised the session. It was interesting to see that the organisation of patient educator sessions had improved over the years:

"So sometimes we didn't know which room we were going into. Uh, and then it would all sort of be in a bit of a hurry. And then we eventually got there, but they were the early days." (Patient 2)

Patient 2, who has been a patient educator for 15 years, then went on to say that in the subsequent years following discussion with the education staff, their experience improved:

"Yeah, yeah, the teaching team, (staff member) and all the rest of them are so organized, it's unbelievable." (Patient 3)

"…there was never a time where I thought ‘what am I meant to be doing, where am I meant to be going?'" (Patient 4)

This is also evidenced by more recent patient educators, Patient 3 and Patient 4, who have been educators for two and three years, respectively. This dialogue with the educators provides confidence that the organisation of the session and communication with each of the educators is currently up to standard.

Motivations for becoming a patient educator

A crucial aspect of this analysis is to delve into what drives patient educators to volunteer and fulfil their roles. There were a couple of overarching themes identified.

Sub-theme 2.1: Wanting to Give Something Back

When questioned about their motivations for becoming a patient educator, a common theme emerged of wanting to *give something back*:

"Well, I think I felt like I was giving something back because I was actually one of the fortunate ones who survived cancer." (Patient 1)

"Because they saved my life. And I was incredibly grateful to the team at The Christie." (Patient 2)

For those patients who have reached a stable point in their cancer journey, there is an indication that they felt indebted to The Christie Hospital and its staff for the care they received. Becoming a patient educator could be interpreted as a way for these patients to *repay* The Christie Hospital by providing a service akin to how a service had been provided for them.

Sub-theme 2.2: Personal Attributes

Motivations also seemed to extend to how each of the patient’s personal attributes could contribute to delivering teaching to students, giving them another sense of purpose. As mentioned previously, Patient 2 believes that their background in advertising was a favourable trait as they were used to being conversational and prompting discussion. Furthermore, Patient 1 explains how being a nurse was advantageous in her approach:

"Being a nurse I've seen lots of doctors at various stages and sometimes they can just see the illness rather than the whole life. So, in my story I would say I was a mother of three young children and I was a nurse.” (Patient 1)

This suggests that from Patient 1’s perspective, their role as a nurse meant they had first-hand experience working with doctors, including observing how they delivered information to patients. The fact that Patient 1 was a health care professional seemed to validate the patient’s standing within the realms of teaching medical students. In another example, Patient 4 believed that their previous role as a secondary school teacher was a key factor in deciding to become a patient educator: 

"I suppose, you know the teacher side of me quite enjoyed being in front of students again, you know, and that's what I miss." (Patient 4)

Patient 4 also cited that one of the reasons they think they were approached to become an educator was because of their teaching background. Furthermore, the patient referenced their teaching career throughout the interview to demonstrate their insight into teaching decisions, such as how the sessions were facilitated and why it was understandable that there were variations in teaching styles depending on the facilitator:

"So, you know, it was good and bad. But like I say, as a teacher, I know each lecturer has different teaching styles." (Patient 4)

These were interesting observations, as most patient educators felt as though they had to validate their role as a patient educator by stating additional personal attributes, which made them suitable for the role. 

Sub-theme 2.3: Improving Experiences of Future Patients

Another key factor motivating patient educators was based on their prior experiences as patients while undergoing treatment. In particular, the more negative aspects of their interactions with health care professionals had a large influence, with some feeling it was their inherent duty to try and improve the experiences of future patients by educating the *doctors of tomorrow*:

“I thought if I could improve it so that someone else had a better experience because of my experience, it will feel a bit more worthwhile.” (Patient 1)

"As I look at it, these junior doctors are my children and grandchildren's future. You know, with cancer it's not giving the medication, it's understanding how a person feels with the disease." (Patient 3)

"I had some really good experiences and I had really bad experiences. So you know, I think it's important for the students to realize what would be good for them to do and also what not to do." (Patient 4)

These comments demonstrate an altruistic motivation for wanting to share their experiences, with emphasis particularly on ensuring health care professionals took a holistic view when treating the patient. Patient 3 regarded it as of great importance for physicians to consider the psychosocial impact of the illness as well as the physical impact. Patient 1 also seemed to share this sentiment, advocating that doctors take a more holistic approach to patient care:

"So I think I was hoping that then they'll be able to see that it wasn't just an illness. There was a whole person behind that illness and maybe it might make them better doctors out of it." (Patient 1)

Overall, there are several interplaying factors contributing to patients’ motivations to become educators, with the sub-themes identifying both altruistic and personal reasons for taking up the role.

Benefits of engaging in medical education

Each of the patients was also asked how they have benefited from engaging in an educator role. The perceived benefits were personal to each patient, although still demonstrated a couple of overarching themes.

Sub-theme 3.1: Improvement in Mental Health

Each of the patients reported a positive impact on their mental health, both implicitly and explicitly, following engagement in teaching sessions as an educator:

"I think it's quite good to reflect on what's happened and how far you've come up in your own journey… when you think ‘that's how I felt then and this is my life now’ it makes you a bit more appreciative of the fact that you've survived and what you went through." (Patient 1)

Patient 1 found that retelling their experience to medical students was a useful way to reflect on how far they had come since their initial diagnosis. Patient 4 also shared a similar view:

"I think it was good personally for me as well, so from a really selfish point of view, because its been eight years since I was ill and it's good to see how far I've come and talking through things." (Patient 4)

This evidences the importance of reflection for patients following their cancer treatment, allowing them to gain perspective on their past tribulations and how they have grown from them:

"I would say it's a kind of destress as well, that you can openly talk about it […] you gotta be careful what you're talking to your family about, because in the back of your mind you don't want to upset them […] by talking to students, they just ask those questions that you want to give answers to, and then you're not causing any upset to the family." (Patient 3)

Patient 3 here offers an interesting insight into how being an educator helped with coping at home. Being able to talk about their journey with people other than their family acts as a healthy outlet, as it takes the burden of distressing memories away from close relatives, instead relaying these to students who may not be as emotionally invested.

Sub-theme 3.2: Engaging With Medical Students

"It's quite a privilege to be able to sort of shape these young doctors, because having worked with older doctors and being a nurse, you think if you could just tell them things now, that would make them better doctors in the future." (Patient 1)

"The fact that I was helping young people become whatever they were aiming for in healthcare, and I thought well if I can help in anyway then that was a good thing." (Patient 2)

Patients believed they benefited by talking to medical students as they felt they were providing a service to both students and the wider community. This closely ties in with their motivations to improve the experiences of patients in the future, as they feel they are shaping and educating the doctors of tomorrow. Some patient educators also mentioned that they found receiving feedback from students who attended their session helpful, reaffirming the effectiveness of their role in the development of medical professionals:

"What's really, really good is, I've seen some comments from the students saying, ‘well, after speaking to Patient 3, I want to go down the oncology route’ […] that's a big lift. Yeah, I think 'Oh I’ve done something there, I'm going to make a good doctor there from them listening to me.'" (Patient 3)

This comment also ties in with the impact on mental health, as Patient 3 mentions they were uplifted by the fact that they had made a difference, perhaps by inspiring students to deeply consider oncology as a career.

Suggested improvements for the session

Despite very positive feedback all around from each of the patient educators, they were also asked if there were any negatives to acting as a patient educator.

Sub-theme 4.1: Logistics 

A common theme from the patient educators were logistical matters, particularly the length of time allocated to the session:

" […] sometimes the question time wasn't quite long enough. Sometimes you could be in really good discussions, but then obviously had to move on to the next stage, so that was a bit frustrating 'cause you knew you could tell them more." (Patient 1)

"So, the only downside is, I think 45 minutes of questions is not enough. Because it takes you half an hour to break the ice with the medical students. And I think once they feel more comfortable with you, then they’ll ask those in-depth questions." (Patient 3)

Patient 3 mentions the importance of being able to *break the ice* with students to allow them to build confidence through the course of the conversation. This may encourage students to ask those more pertinent and searching questions that they may not have done at the start of the sessions. A fine balance, therefore, needs to be struck between allowing time to settle into the conversation while also ensuring that the student’s intended learning objective are covered appropriately within their timetabled teaching.

Sub-theme 4.2: Recruitment

"One of my research nurses when I was on the clinical trial […] knew I was a teacher, or an ex-teacher, and just literally walked past me as he was going to wherever he was going and told me about it. It was all sort of very adhoc, I suppose." (Patient 4)

Each of the patient educators interviewed reported being recruited to the teaching programme with a seemingly impromptu approach. These educators were asked by either doctors or nurses in a clinic setting unrelated to medical education or in happenstance while walking along the hospital corridor. Although this is not necessarily a drawback for these educators when asked about how we could improve their experience, Patient 4 went on to say:

"How you enroll people I suppose, like I said, it was just a chance meeting with me, otherwise I wouldn't have known anything about it." (Patient 4)

This suggests that Patient 4 recognises other patients may not be given the same opportunity to become an educator and benefit from the experience in the same way that they have done. It is important to mention, however, that each patient educator finalised the interview stating that they enjoyed their roles and would love the opportunity to continue providing this service for students.

## Discussion

This study investigated cancer patient educators’ experiences within medical education, exploring four key themes. The superordinate themes yielded by the IPA consisted of educators’ perceived success of the session, motivations for becoming a patient educator, perceived benefits of engaging in medical education and suggested improvements for the session. 

Perceived Success of the Session

Three subthemes were identified by patient educators’ for how they perceived the success of the session. The first subtheme was the relationship between themselves and the facilitator of the session. The role of the facilitator within the teaching session appears to be mixed, and this is mainly due to inconsistency in tutors leading the session. As evidenced in the IPA, it appears that facilitators that took more of a *back-seat* role, allowing the conversation to play out with minimal interference, seemed to improve patient educators’ experience. Those tutors who *took over* the session made the patient at times feel redundant, in effect using the patient as a tool to deliver a teaching session rather than as an authentic narrator whose experience the session should be based around. The role of the facilitator is important as his behaviour influences patients’ experiences. Having a good relationship with the patients, being committed to the teaching programme, making them part of the teaching and not objectifying them, enhances the learning experience for both students and patients [14,15]. 

The second sub-theme identified was students’ willingness to participate in the discussion. This is a key factor in the success of any teaching session centred around maintaining a dialogue with a patient. The willingness of students to participate in the discussion is also interlinked with other subthemes, such as personal attributes, where educators believe that due to their unique background/skillset, they could encourage students to participate in the conversation. Furthermore, regarding the logistics subtheme, educators thought that more time was needed to break the ice with students, which again lead to more open and honest discussions.

Finally, the organisation and planning of the session were crucial to ensure the educator understood the format of the session, as well as where they were meant to be on the day. Most patients mentioned that they were well informed by clerical staff in the days leading up to the session and were highly complementary of the medical education team. This first superordinate theme has therefore demonstrated we are developing quite a complex system of relationships between the staff organising, the host, the students, and the educator. The patient educator is an equal partner in those relationships.

Motivations for Becoming a Patient Educator

A common subtheme identified is patient educators showing gratitude for their care and wanting to *give something back*. This is a consistent finding in the literature, with several articles citing patients who want to be involved in medical teaching so they can repay the care they were provided while being ill [3,16-19]. The educators’ altruism has two components: one aimed at the hospital and staff where they have received care and a wider one where patients believe being involved in teaching medical students will provide enhance and broaden students’ experience, influence practice and help the wider community. This is linked with the other subtheme explored further down, as the educators believe sharing their stories and experience will lead to a better service for future patients.

The second subtheme relates to the patients themselves and how their attributes make them suitable for being educators. Our patients come from different backgrounds (nursing, teaching and advertising), so they interact with students differently. All of our patients stated that their previous work experience has helped them in engaging with students, and they used this to validate their suitability for the role. Taking a closer look at this, we can associate the patient’s work background with certain skills they have developed. A nurse has more insight when talking with health care professionals or discussing a medical condition, while a teacher has to be approachable and with good communication skills. In a study looking at what attributes a good educator should have, as seen by second-year medical students, communication, approachability and willingness to help were the qualities students were looking for in an educator [20].

Finally, the educators are looking at improving the experience of future patients by sharing their own experiences and challenges encountered. By talking about some of the bad or unpleasant encounters they had, educators hope to make doctors understand their struggles so that future patients don’t go through the same issues. At the same time, educators reinforce the idea of patient-centred care as they should be seen as a whole and not just as a disease that needs to be treated [3,21,22]. 

Perceived Benefits of Engaging in Medical Education

One of the main benefits of engaging in medical education that we found in our study was that the educators acknowledged an improvement in their mental health. As they were sharing their stories, they had the chance to reflect on their experience: it allowed them to be grateful for how far they have progressed since they were diagnosed, how to cope with cancer and get a deeper understanding of their illness. In a study looking at the role of gratitude in breast cancer patients, it was revealed gratitude is associated with post-traumatic growth (positive life changes after trauma), increased positive emotions and reduced distress [23]. In a more comprehensive study, examining 30 cancer narratives published by three U.S. cancer centres, cancer survivors are shown to share their stories as a coping mechanism and as a way to express gratitude towards the cancer centre, which is seen as a saviour [24]. This relates to our previous subtheme, where the sense of altruism is associated with *giving back* to the hospital where patients were treated. 

The other subtheme identified is engagement with medical students. This is important as educators feel it helps not only the students but also future patients, bringing it into relation with one of the previous subthemes discussed. By telling their story, patients felt they were making an impact on the students, educating and inspiring them. This is a common finding in literature as engagement with students was felt to be an overall a positive experience for both the educators and students [2,5,22]. Involving patient educators in medical education gives an authentic insight into how living with cancer affects their lives. Students have the chance to listen to them, get to understand both the visible and invisible impact of a chronic condition and recognise patients are people first. This promotes patient-centred care as it makes patients active participants and encourages students (and future doctors) to be more mindful and empathic towards the patient’s needs. 

Suggested Improvements for the Session

Although there has been positive feedback regarding the PAT sessions, educators noted improvements are needed when it comes to the length allocated to the session as time is required for students to *break the ice*. This is important as students can become more confident and ask relevant questions, and the educators would have enough time to share their stories. For future sessions, we are looking to increase the allocated time from 45 minutes to one hour so that a balance is struck between the educators’ desire to not feel rushed during the interaction with the students and the proper coverage of the student’s intended learning objectives.

Another issue raised by the educators was how they were recruited, stating it was more of an ad hoc approach, during a meeting unrelated to teaching, rather than a formal one. For future sessions, contacting potential patient educators via e-mail would ensure the process is a more formal one.

## Conclusions

Our study has shown what motivates patients to become educators and how it benefits them. Patients are motivated to become educators because they feel grateful for the care they have been provided and want to repay this by sharing their experience with medical students in the hope that this will make them better doctors. Also, this altruism extends to future patients as educators think about other patients who will be in the same situation as them. We have also shown that being involved in medical education improves cancer patients’ mental health as reflecting on their journey from cancer diagnosis to cure made them grateful and helped their families to cope with the situation. At the same time, a balance must be achieved between the educators’ eagerness to share their stories and ensuring the students achieve their intended learning objectives. Although in previous studies patient educators were described as affected by chronic health issues (diabetes, chronic pain, stroke, multiple sclerosis or cancer) with no difference between them, our study focuses exclusively on patient educators with a cancer diagnosis and their experience in medical education. As far as we know, there is no current evidence in the literature looking only at oncological patient educators and corroborated with the overall under-representation in the literature of what motivates patient educators to take part in medical education. We believe our study to be a valuable contribution to this domain.

There are several limitations to our study. First, this was a study done with a small number of participants. Although IPA is a methodology best used on a small number of people as it provides a more in-depth analysis, interviewing more patients could have uncovered other themes and sub-themes apart from the ones discussed. Second, all of our patients have been in remission from cancer, so their shared experience could have felt ordinary to them. For future research, it would be interesting to see if similar or maybe different findings are reported in patients with active cancer. Finally, as we believe this is the first time someone has studied only oncological patients involved in medical education, we do not claim our findings to be universally valid, but they add another layer to the wider discussion of engaging patients in medical education.

## Figures and Tables

**Figure 1:**
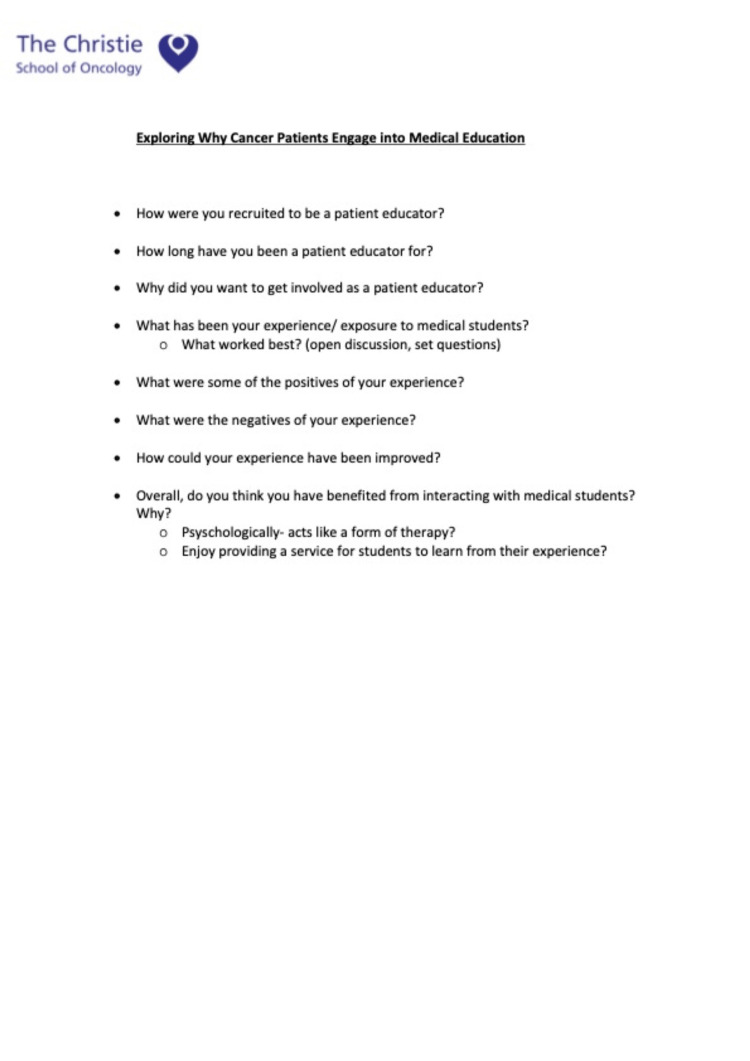
Interview questions proforma.
